# HeLM: a macrophyte-based method for monitoring and assessment of Greek lakes

**DOI:** 10.1007/s10661-018-6708-1

**Published:** 2018-05-05

**Authors:** Dimitrios Zervas, Vasiliki Tsiaoussi, Ioannis Tsiripidis

**Affiliations:** 1 The Goulandris Natural History Museum - Greek Biotope / Wetland Centre, 14th km Thessaloniki-Mihaniona, Thermi, P.O. Box 60394, 57001 Thessaloniki, Greece; 2 Department of Botany, School of Biology, Aristotle University of Thessaloniki, GR-54124 Thessaloniki, Greece

**Keywords:** Macrophytes, Greek lakes, Ecological status, Reference conditions, Class boundaries, Water Framework Directive

## Abstract

The Water Framework Directive (WFD) requires Member States to develop appropriate assessment methods for the classification of the ecological status of their surface waters. Mediterranean region has lagged behind in this task, so we propose here the first developed method for Greek lakes, Hellenic Lake Macrophyte (HeLM) assessment method. This method is based on two metrics, a modified trophic index and maximum colonization depth *C*_max_ that quantify the degree of changes in lake macrophytic vegetation, as a response to eutrophication and general degradation pressures. The method was developed on the basis of a data set sampled from 272 monitoring transects in 16 Greek lakes. Sites from three lakes were selected as potential reference sites by using a screening process. Ecological quality ratios were calculated for each metric and for each lake, and ecological status class boundaries were defined. For the evaluation of effectiveness of the method, the correlations between individual metrics and final HeLM values and common pressure indicators, such as total phosphorus, chlorophyll *a* and Secchi depth, were tested and found highly significant and relatively strong. In addition, the ability of HeLM values and its individual metrics to distinguish between different macrophytic communities’ structure was checked using aquatic plant life-forms and found satisfactory. The HeLM method gave a reliable assessment of the macrophytic vegetation’s condition in Greek lakes and may constitute a useful tool for the classification of ecological status of other Mediterranean lakes.

**Electronic supplementary material:**

The online version of this article (10.1007/s10661-018-6708-1) contains supplementary material, which is available to authorized users.

## Introduction

The Water Framework Directive (WFD; European Commission 2000) supervises the monitoring and assessment of the ecological status of surface waters within the EU. One of the main aims of the Directive is the development of ecological assessment methods for different groups of organisms (biological quality elements (BQEs), i.e., phytoplankton, macrophytes and phytobenthos, macroinvertebrates, fish) for aquatic ecosystems (lakes, rivers, transitional waters, and coastal waters) (European Commission 2003a). These methods need to classify the ecological status of surface waters at a five-level ecological classification scheme, indicating the degree of deviation from reference conditions (European Commission 2003a).

Aquatic macrophytes are commonly used in classification systems in lakes as they play a significant role in determining the structure and functions of lake ecosystems, by altering environmental conditions, nutrient cycling, biotic assemblages, and interactions (Engelhardt and Ritchie 2001; Spoljar et al. 2017; Wang et al. 2009). Moreover, their time of response to changes in nutrient conditions in a water body lies between the fast-responsive phytoplankton and phytobenthos organisms and the slow-responsive invertebrates and fish, which makes them valuable bioindicators, when monitoring the ecological quality of water bodies (Pall and Moser 2009; Penning et al. 2008a, b; Schneider and Melzer 2003). Sunlight penetration and availability are important factors for the development of submerged macrophytes and explain greater than half of the variation in macrophyte composition and abundance (De Boer 2007). Loss of macrophytic vegetation has been experienced in many lakes around the world during the last century due to the increase in the availability of nutrients in the water column that led to a rapid growth of phytoplankton, which in turn caused an increase in water turbidity and a decrease in sunlight penetration and availability (Blindow et al. 2006; Hilt et al. 2006).

After the publication of WFD, most Member States (MS) have focused efforts to develop WFD-compliant aquatic macrophyte methods to assess the ecological status of lakes (Birk et al. 2012; Poikane et al. 2015). However, there are still gaps in the Mediterranean region, as a reflection of different monitoring traditions in comparison to Central and Northern European countries (Birk et al. 2012). More specifically, with regard to natural lakes, MS in the Mediterranean region encountered difficulties in the development of their ecological assessment methods due to the small number and high variability of natural lakes, as well as the limited data availability (Poikane et al. 2015). Another important reason for this time-lag in the monitoring and assessment research in the Mediterranean region is the substantial differences between warm Mediterranean lakes and colder temperate ones (Alvarez Cobelas et al. 2005; Beklioglu et al. 2007). The Mediterranean climate is characterized by a strong seasonality of rainfall and air temperature, whereas rainfall occurs mostly in spring and autumn (Alvarez Cobelas et al. 2005). According to Hoerling et al. (2012), the land area surrounding the Mediterranean Sea has experienced 10 of the 12 driest winters since 1902 in just the last 20 years. Correspondingly, water shortages and droughts are not uncommon in many Mediterranean areas. As a result, Mediterranean lakes experience continuous intra-annual and inter-annual water-level fluctuations which render them a type of aquatic ecosystems under constant alterations (Özen et al. 2010). All the above add to the necessity for the development of a complete lake assessment method based on aquatic macrophytes for the Mediterranean region.

In this study, we overcome this gap and we present the newly developed ecological assessment method named as Hellenic Lake Macrophyte assessment method (HeLM) for classification of Greek lakes. In particular, the development of this method was grounded in (a) the collection of macrophyte and environmental data from natural lakes of the Greek National Water Monitoring Network, (b) the definition of type-specific reference conditions for aquatic macrophytes, (c) the use of appropriate metrics to calculate the deviation of each lake’s ecological status from reference conditions, and (d) the setting of status class boundaries. The result is an assessment method that can address changes in the ecological status of lakes due to eutrophication and general degradation pressures, in compliance with WFD requirements.

The aims of this study are (a) to describe the rationale and the application of the HeLM method, (b) to test its effectiveness to assess the ecological status of Greek natural lakes, and (c) to discuss its limitations and needs for further improvements in the future.

## Materials and methods

### Study area

**Fig. 1:**
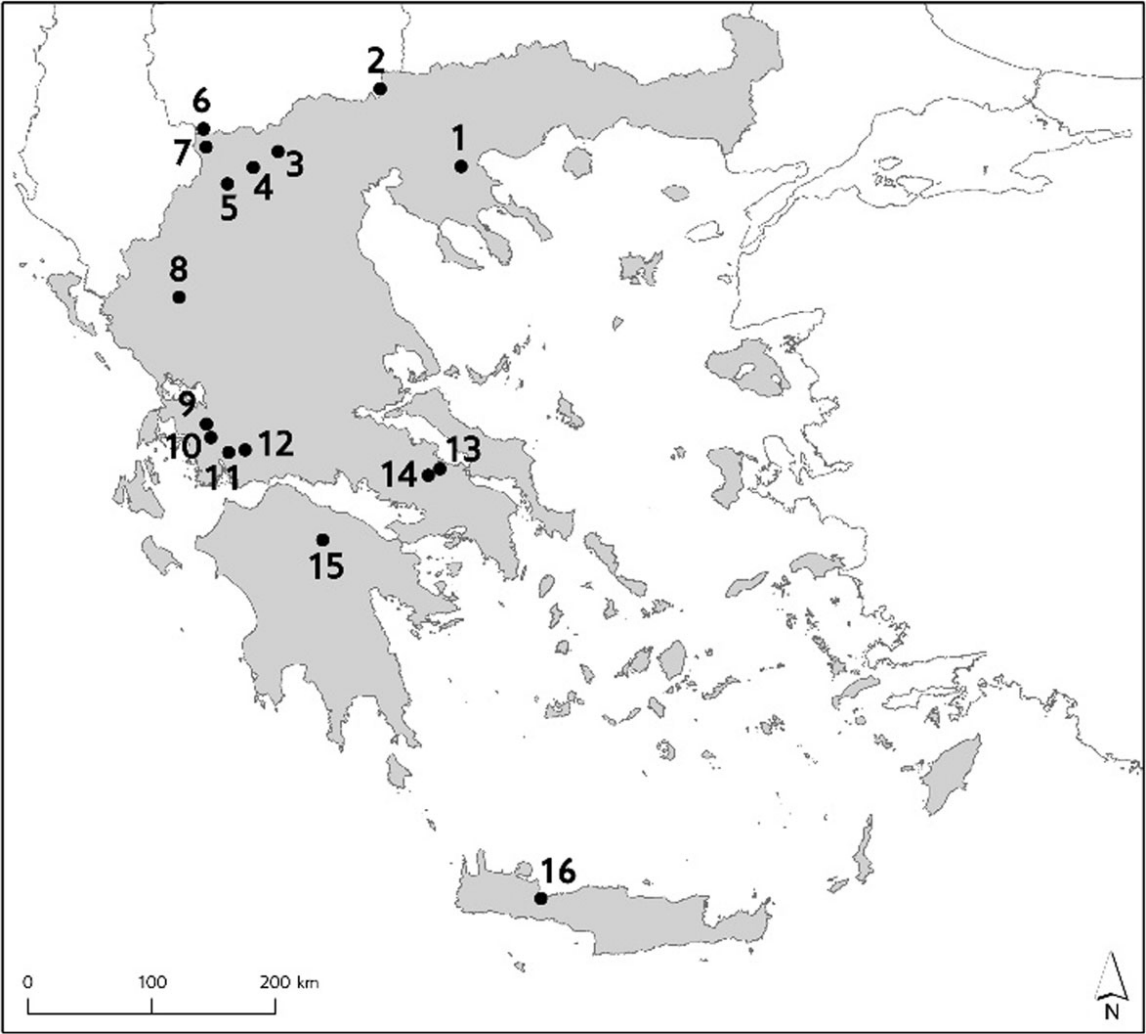
Lakes in the Greek National Water Monitoring Network for which macrophytic data were acquired. See Table 1 for lake names

The Greek National Water Monitoring Network became operational in 2012. In total, 50 lake water bodies (including 24 natural lakes and 26 reservoirs) have been included in the network. Sixteen lakes have been monitored for aquatic macrophytes during 2013–2015 (Fig. 1 and Table 1). According to ETC/ICM (2015), they all belong into the Mediterranean lake types. In particular, eight of them are warm monomictic, deep natural lakes with mean depth > 9 m (national type GR-DNL) and eight are polymictic, shallow natural lakes with mean depth 3–9 m (national type GR-SNL). From the eight GR-DNL type lakes, Feneos lake (no. 15) is a reservoir constructed for irrigation purposes. However, since the irrigation network has not been constructed, water level fluctuates only due to natural conditions; therefore, a species-rich and abundant aquatic vegetation has been developed to such an extent that lake Feneos resembles ecologically a natural lake.

**Table 1:** Sampling period and number of transects established for the investigation of the macrophytic vegetation, in the 16 lakes of the Greek National Monitoring Network

No.	Lake	Abbreviation	Type	No. of transects	Sampling period
1	Volvi	VOL	GR-DNL	20	September 2013
2	Doirani*	DOI	GR-SNL	10	August 2013
3	Vegoritida	VEG	GR-DNL	20	August 2013
4	Zazari	ZAZ	GR-SNL	12	August 2015
5	Kastoria	KAS	GR-SNL	20	August 2014
6	Megali Prespa*	MEP	GR-DNL	12	August 2015
7	Mikri Prespa*	MIP	GR-SNL	15	August 2015
8	Pamvotida	PAM	GR-SNL	20	September 2013
9	Amvrakia	AMV	GR-DNL	20	June 2014
10	Ozeros	OZE	GR-SNL	20	June 2014
11	Lysimachia	LYS	GR-SNL	20	June 2014
12	Trichonida	TRI	GR-DNL	20	July 2015
13	Paralimni	PAR	GR-SNL	19	July 2014
14	Yliki	YLI	GR-DNL	20	July 2014
15	Feneos	FEN	GR-DNL	10	August 2014
16	Kourna	KOU	GR-DNL	14	May 2014
Total		16 lakes	2 types	272	3-year period

GR-DNL type are warm monomictic, deep natural lakes with mean depth > 9 m while GR-SNL type are polymictic, shallow natural lakes with mean depth 3–9 m. Transboundary lakes that only the part of their surface area in Greece was surveyed are marked with asterisks

### Sampling

In all 16 lakes, the belt transect-mapping method was applied. It is the most commonly applied method for aquatic vegetation surveys and monitoring in many European countries and it is also recommended by the European Committee for Standardization (CEN), as it provides at the same time abundance, frequency, and depth distribution data of different species in a lake (Kolada et al. 2009).

The number and location of transects were selected using the Jensen’s method (1977), bathymetric data, habitat maps, and land use maps for the lakes and their catchment area. Four different groups of riparian habitat types were distinguished around each lake, using the classification scheme of XP T90–328 (2010) Standard. In each group, at least three transects were established. This number was increased in cases of high variability (e.g., ecological, morphological, hydrological reasons) within each group of riparian habitat types. The final number of transects in each lake ranged from 10 to 20. In total, 272 transects were established (Table 1).

Sampling within each transect followed the guidelines proposed by I.S. EN 15460 (2007) and Kolada et al. (2009). Transects were perpendicular to the lake shoreline and represented a strip area from the shoreline to the maximum depth of plant growth. The strip area had a width of ca. 5 m to enable boat maneuvering and the handling of the sampling tools. Sampling was conducted in five depth zones: 0–1, 1–2, 2–4, 4–8 m, and > 8 m (Janauer 2002) by means of a double-headed rake with a scaled handle or attached to a rope, a bathyscope, and a geo-bathymetric device. In each depth zone, five plots, evenly distributed along the increasing depth gradient, were sampled. All angiosperms (helophytes, hydrophytes, amphiphytes, and aquatic forms of land species), pteridophytes, bryophytes, charophytes, and other green filamentous macroalgae (e.g., *Cladophora* spp.) were recorded in each plot, and their abundance was estimated by using the semi-quantitative five-point DAFOR scale (Palmer et al. 1992). Angiosperms, pteridophytes, bryophytes, and charophytes were determined to the species or subspecies level by using suitable floras and identification keys (Online Resource, Supplement 1).

Furthermore, for each transect, the maximum colonization depth of aquatic macrophytes (*C*_max_) was recorded. To ensure its proper measurement, at the end of each transect, more than one plots with no vegetation were sampled. At each lake, three transects with larger *C*_max_ values were revisited annually during the 3-year period. Thus, 36 transects were revisited, and in total, 308 measurements of *C*_max_ at transect level were made.

Environmental data indicating eutrophication pressure were also collected during the 3-year sampling period. In each lake, two to three monthly samples for chlorophyll *a* (CHLA) and Secchi depth (SD) measurements were taken during each summer season (June to August). Samplings for total phosphorus (TP) were seasonal for each year. Water samples were taken at the deepest part of the lakes, from the euphotic zone (2.5 × SD depth) by using either an integrated-type or a Nansen-type sampler (de Hoyos et al. 2014). TP was determined with persulfate digestion (Rice et al. 2012) and CHLA by using 90% acetone and applying the trichromatic equation (Rice et al. 2012; Jeffrey and Humphrey 1975). Land use data for the catchment area of each lake was acquired by Corine Land Cover (CLC) 2012, version 18.5.1 (Copernicus Service–Pan-European Component 2012). Population data were acquired by the 2011 Population-Housing Census (Hellenic Statistical Authority 2011).

### Development of the HeLM assessment method

#### Rationale for metric selection

According to WFD requirements, ecological status assessment based on aquatic macrophytes should take into consideration both their taxonomic composition and abundance (European Commission 2000). Existing taxonomic composition metrics vary from simple ones, such as diversity indices (total number of taxa or number of submerged taxa, etc.), proportions of different functional groups of species (relative coverage of charophytes, isoetids, etc.), to more complex ones based on each species’ sensitivity to disturbance represented by scores, such as trophic indices, indicator species, and sensitive/tolerant taxa (Birk 2010; Birk et al. 2012; Poikane et al. 2015). For the measurement of abundance, the most widely used metric is *C*_max_, which simply expresses the maximum observed depth of a lake where submerged rooted macrophytes are present (Birk 2010; Poikane et al. 2015). *C*_max_ is considered as a very useful measure of total macrophyte abundance in lakes, being tightly connected to water transparency and thus to trophic state (Pall and Moser 2009). It responds quickly to changes of water quality (Mehner et al. 2008) and its assessment is quite unbiased (Willby et al. 2009). *C*_max_ values can range from zero meters in hyper-eutrophic lakes with no submerged aquatic vegetation to many meters of depth in oligotrophic lakes with extensively developed submerged vegetation. In very shallow lakes, where there is not a depth limit of macrophytic growth, the relative mean percent of macrophyte coverage of total lake area is used instead (Birk 2010; Kolada 2014; Poikane et al. 2015).

The response of the most common macrophyte metrics in eutrophication gradients in lakes was explored and tested by Dudley et al. (2011), Kolada et al. (2011), and Kolada et al. (2014). Their results signified that indices based on trophic scores perform better, with the Intercalibration Common Metric for lake macrophytes (ICM_LM_) being the best performer, followed by Ellenberg Index (EI). Species richness metrics (number of taxa and number of submerged taxa) responded weakly to eutrophication gradients, while the metrics based on proportions of functional groups responded differently in ecosystems of lower and higher trophy, weakening their diagnostic value. For the abundance metrics, it was found that both *C*_max_ and the coverage of aquatic macrophytes respond significantly to eutrophication stressors. The former metric was recommended for use in lakes with mean depth > 3 m and the latter in very shallow lakes (mean depth < 3 m) (Kolada et al. 2011; Kolada 2014).

During the development of the HeLM assessment method, we tested the above-mentioned metrics in various combinations, for their response to eutrophication pressure with data from the Greek Monitoring Network. We found that ICM_LM_ and *C*_max_ were the best-performing ones; thus, HeLM assessment method was designed using these two metric components.

#### Trophic index HeLM

The trophic index metric HeLM (TIHeLM) we applied is a modified form of ICM_LM_, which is based on taxon-specific trophic scores (lake trophic ranks (LTRs)), originally developed for the purpose of the WFD intercalibration exercise for lakes (Hellsten et al. 2014; Kolada et al. 2011, 2014). ICM_LM_ had to be modified in order to be more effective in evaluating the eutrophication pressure in Mediterranean-type lakes. Three modifications were made concerning the calculation of (a) the LTRs for taxa not included in Kolada et al. (2011, 2014), (b) the weighted average of LTRs per transect by using species cover as weight, and (c) the trophic index of each lake by averaging the corresponding index values of the transects sampled in the lake.

In Kolada et al. (2011, 2014), there were LTR scores for 43 taxa out of the 92 recorded in the 16 Greek lakes. Most of the taxa not included were helophytes, which in some cases were the only representatives of macrophytic vegetation in eutrophic and degraded lakes. These taxa provide reliable information on ecosystem ecological conditions and can support assessment of the ecological status of lakes under eutrophication pressure (Alahuhta et al. 2012; Kolada 2014, 2016). Moreover, the inclusion of helophytes is recommended since the assessment of eutrophication seems to be more reliable when more scored taxa are considered (Kolada 2014; Kolada et al. 2011). So, following the method used by Kolada et al. (2011, 2014), the missing LTRs were estimated from the regression equation between LTR values and the indicator values of Ellenberg for nutrients N (Ellenberg 1988; Ellenberg et al. 1991): *LTR* = 1.395*N* − 0.6276 (*R*^2^ = 0.64, *n* = 98, *p* < 0.0001). At the current state, LTR scores for macrophytic taxa, as calculated during the pan-European intercalibration exercise, were used due to lack of available data for the development of a Mediterranean or a Greek specific taxa list. The other two modifications were made to optimize the ICM_LM_ metric. Cover-abundance values and metric calculations per transect are commonly used in calculations of trophic indices in other MS assessment methods (Hellsten et al. 2014; Pall et al. 2014; Portielje et al. 2014). For the calculation of the weighted average of LTR values per transect, the LTR value of each taxon was multiplied with its relative abundance within the transect. The latter was calculated after transforming the ordinal values of DAFOR scale to percentage cover values on the basis of the following correspondence: dominant = 87.5%, abundant = 50%, frequent = 17.5%, occasional = 5.5%, and rare = 0.5%, and raising the percentage cover values to the power 0.2, to avoid a high weight of dominant taxa in the index. Finally, a TIHeLM value for each lake was calculated by averaging the TIHeLM values of the transects sampled in the lake.

#### Maximum depth of colonization (*C*_max_)

All 16 lakes in the current data set have mean depth above 3 m, so *C*_max_ was chosen as the best available macrophytic abundance metric (Kolada 2014; Kolada et al. 2011, 2014). As Kolada et al. (2011, 2014) remarked, *C*_max_ is subject to annual variations, which should be taken into account to reduce the risk of misclassification of a lake. For that reason, the value that we used in the HeLM assessment method for each lake was the mean average of annual *C*_max_ values measured in the 3-year period.

#### Establishment of type-specific reference conditions

Establishment of reference conditions for BQEs is crucial to ecological assessment of surface water bodies, as their ecological status is determined by their degree of deviation from these conditions. Sites in reference conditions do not equate to water bodies in pristine state. They are defined as those expected in natural or near-natural state, with no or minimal disturbance and with human pressure resulting in minor effects on biological elements (European Commission 2003b).

Reference conditions for HeLM assessment method were based on existing near-natural reference sites, with the application of appropriate pressure criteria (e.g., European Commission 2003b; Poikane et al. 2015):

- Total phosphorus concentration (TP) calculated as mean annual value,
- Chlorophyll *a* concentration (CHLA) calculated as mean summer value (June–August),
- Secchi depth (SD) calculated as mean summer value (June–August),
- Artificial land use (ALU), composed of the sum of percent cover of all the categories of CLC belonging to class 1 (urban areas continuous and discontinuous, industrial and commercial zones, communication infrastructures and networks, mines, etc.) in the catchment area,
- Intensive agriculture (IA), composed of the sum of percent cover of CLC categories corresponding to a high potential impact from agricultural activities (arable and irrigated land, permanent and annual crops, vineyards, orchards, olive groves, complex cultivation patterns; CLC codes 2.1, 2.2, 2.41, 2.4.2) in the catchment area,
- Natural and semi-natural land use (NASN), composed of the sum of percent cover of forest and natural areas, wetlands, and water bodies (CLC codes 3.1.1, 3.1.2, 3.1.3, 3.2, 3.3, 4, and 5) in the catchment area,
- Population density (PD), calculated as inhabitants per square kilometer (h/km^2^) in the catchment area.

Many of these pressure criteria may be correlated strongly to each other, but applying all of them simultaneously is expected to give a better filtering of low-impacted and thus potential reference sites. For each one of them, a threshold value has been determined (Table 2), for accepting or rejecting a site as a potential reference one, similar to the ones set in Mediterranean MS (e.g., Pahissa et al. 2015). If a site fails even in one of these pressure criteria, then it cannot be considered as a reference site.

**Table 2:** Pressure criteria and their threshold limits established for screening potential reference sites, following the results from Pahissa et al. (2015)

National lake type	TP (μg/L)	CHLA (μg/L)	SD (m)	ALU (%)	IA (%)	NASN (%)	PD (h/km^2^)
Deep natural lakes (GR-DNL)	< 12	< 2	> 6	< 4	< 25	> 70	< 30
Shallow natural lakes (GR-SNL)	< 15	< 5	> 2	< 4	< 25	> 70	< 30

After the initial screening on the basis of pressure criteria, an extra step of filtering was applied, in order to disqualify sites that deviate significantly (outlier values) from those expected under reference conditions. Such deviations could be found for sites with no apparent anthropogenic pressures, but with restricted macrophyte development due to extreme natural landscape parameters (e.g., substrate restrictions, extreme inclination). Sites that qualified for both stages of the screening process were considered to represent reference conditions and were used in the ecological status class boundary setting procedure.

#### Ecological status class boundary setting procedure

Reference values and ecological status class boundaries for TIHeLM and *C*_max_ were calculated as recommended by REFCOND (European Commission 2003b). For TIHeLM, common class boundaries for both national lake types (GR-DNL and GR-SNL) were established, since taxonomic composition was not affected by their difference in maximum depth (there were no deep lake specific taxa and no more depth zone divisions after 8 m of depth). Different class boundaries were calculated for *C*_max_, since the potential maximum colonization depth in GR-SNLs is limited by their maximum depth, in contrast to GR-DNLs which do not have this limitation.

Reference values for both TIHeLM and *C*_max_ metrics were determined as the median values in the selected reference sites. Subsequently, each lake’s metric values were transformed to ecological quality ratios (EQRs) by dividing them by those reference values. In order to determine a high/good (H/G) class boundary for each metric, the 90th percentile (P90) of the distribution of its values in the selected reference sites was used (European Commission 2003b). To determine good/moderate (G/M) class boundaries, we followed the resulting TP range adopted for the Mediterranean Assessment System for Phytoplankton NMASRP (de Hoyos et al. 2014; Pahissa et al. 2015). We also assessed this range’s relevance with our data by checking the changes in aquatic plant life-forms predominance and composition/abundance metrics along the eutrophication gradient. This TP range was found to correspond to the transition point from submerged-dominated to helophyte-dominated macrophytic communities (Online Resource, Supplement 2) and to the space before the metrics cross-over point (which is associated with moderate ecological status sites) in a paired metric analysis between TIHeLM, *C*_max_, and TP (Online Resource, Supplement 3). Thus, G/M class boundaries were determined at 75th percentile (P75) of the distribution of each metric’s values, in sites that belong to the 20–50-μg/L TP group (de Hoyos et al. 2014). For the remaining class boundaries, the EQRs ranging from below the G/M class boundary to their minimum values were equally divided to form the moderate/poor (M/P) class boundary and poor/bad (P/B) class boundary (European Commission 2003b).

#### Final calculation of EQR for each lake and assessment of its ecological status

For the combination of the two metrics (TIHeLM and *C*_max_) in a final value for each lake, a normalization procedure of each lake’s EQR values was applied following piecewise linear interpolation (Hazewinkel 2002) between each status class’ upper and lower boundary value. This implied the conversion of each metric’s EQR to a normalized scale with equal class widths and standardized class boundaries, where the high-good (H/G), good-moderate (G/M), moderate-poor (M/P), and poor-bad (P/B) boundaries were at 0.8, 0.6, 0.4, and 0.2, respectively.

The final lake assessment was determined using the principle of equal weight for taxonomic composition and abundance metrics. Thus, following the calculation of EQRs for both metrics and their normalization procedure for each lake, a final HeLM score and its subsequent ecological status class were calculated by their average.

### Statistical assessment of method’s performance

In order to evaluate the performance of the HeLM assessment method addressing eutrophication and general degradation pressures, the pressure-response relationships were investigated. The pressure indicators used for the evaluation of the metrics were TP, CHLA, and SD. By means of linear regression analysis and multivariate regression analysis (Legendre and Legendre 1998), the relationships between the three pressure indicators and the two metrics of the method separately as well as the final values of the HeLM method were determined.

To improve data distribution, TIHeLM metric values were log-transformed, while *C*_max_ metric values were square-root-transformed. The transformation of HeLM final values did not improve the distribution; thus, the values remained untransformed. Pressure indicator values were all log-transformed. For linear relationships, a linear regression model was applied using IBM SPSS Statistics v.23 (IBM 2014) software, and the resulting coefficient of determination (*R*^2^), Pearson’s correlation coefficient (*R*), and *p* value (*p*) of the model were assessed. As proposed by Kolada et al. (2011), the values of the coefficients *R*^2^ > 0.30 and *R* > 0.55, for statistically significant models (*p* < 0.05), were assumed as sufficient to accept a metric as a well-performing one. For the relationship between HeLM and all three pressure indicators, a multivariate regression model was applied and the same coefficients were assessed using the same software.

Finally, relative abundance values of different life-forms of the macrophytic vegetation (elodeids, helophytes, charids, ceratophyllids, nymphaeids, and lemnids) were calculated for each lake. These values were plotted against the calculated metric values for each lake and polynomial adjustments were applied using IBM SPSS Statistics v.23 software (IBM 2014), in order to quantify the responses of different groups of aquatic macrophytes along the eutrophication gradient.

## Results

### Development of the HeLM assessment method

In total, 92 macrophyte taxa were recorded in 16 Greek lakes. The lists of these taxa along with their LTR values for the calculation of TIHeLM are given in Online Resource, Supplement 4.

Based on the environmental data used as pressure indicators, three lakes were selected as potential reference ones: Kourna and Feneos (GR-DNL type) and Paralimni (GR-SNL type) (Table 3). The difference in the distribution of the pressure criteria values between non-reference and reference lakes is shown in Online Resource, Supplement 5. For these three lakes, 41 out of the 43 sampled transects were qualified as reference on the basis of the macrophytic vegetation and they were selected to represent reference conditions. Following the setting of reference conditions, type-specific ecological status class boundaries were calculated for each metric (Table 4).

**Table 3:** The lakes selected to represent reference conditions, after the pressure screening process, with their calculated values for the selected indicators of eutrophication and general degradation pressure

Lake	Type	TP (μg/L)	CHLA (μg/L)	SD (m)	ALU (%)	IA (%)	NASN (%)	PD (h/km^2^)
Kourna	GR-DNL	< 10	1.27	7.5	0.00	2.70	97.30	6.68
Feneos	GR-DNL	10.27	0.47	9.9	0.00	0.00	95.47	0.00
Paralimni	GR-SNL	13.63	3.78	2.5	0.16	22.24	77.59	9.57

For the pressure indicator abbreviations, see Materials and methods / Development of the HeLM assessment method / Establishment of type-specific reference conditions Section

*GR-DNL* Greek deep natural lakes, *GR-SNL* Greek shallow natural lakes

**Table 4:** The type-specific ecological status class boundaries (ecological quality ratio and raw values) as calculated for each metric of the HeLM assessment method

Metric	TIHeLM		*C*_max_ (m)		*C*_max_ (m)	
National lake types	GR-DNL and GR-SNL		GR-DNL		GR-SNL	
Class	EQR	Value	EQR	Value	EQR	Value
Reference	1	7.14	1	12.2	1	6.1
High	> 0.94	< 7.60	> 0.89	> 10.86	> 0.69	> 4.21
Good	0.94–0.90	7.60–7.93	0.89–0.36	10.86–4.39	0.69–0.58	4.21–3.54
Moderate	< 0.90–0.82	> 7.93–8.71	< 0.36–0.24	< 4.39–2.93	< 0.58–0.39	< 3.54–2.38
Poor	< 0.82–0.75	> 8.71–9.52	< 0.24–0.12	< 2.93–1.46	< 0.39–0.19	< 2.38–1.16
Bad	< 0.75	> 9.52	< 0.12–0	< 1.46–0	< 0.19–0	< 1.16–0

*GR-DNL* Greek deep natural lakes, *GR-SNL* Greek shallow natural lakes

### HeLM method application

The results of the calculations of TIHeLM and *C*_max_ for the 16 Greek lakes (raw values and normalized ecological quality ratios), the final HeLM values, and the consequent ecological status class for each lake are given in Table 5. Twelve out of the 16 lakes were classified at high and good ecological status. The remaining four were positioned in the lower classes. In six lakes (Kourna, Feneos, Trichonida, Paralimni, Lysimachia, and Zazari), both metrics independently resulted in classifying them in the same ecological status class. For the other 10, the two metrics gave results that differ up to two classes of ecological status. TIHeLM values exhibited a similar variance among the transects within each lake (Online Resource, Supplement 6). An obvious exception was Lysimachia lake (LYS), in which half of its transect showed a high level of degradation, whereas the other half did not.

**Table 5:** Calculated values for metrics TIHeLM and *C*_max_ of the HeLM assessment method (raw values and normalized ecological quality ratios (nEQRs)) and final assessment of the ecological status for each one of the 16 lakes of the Greek National Water Monitoring Network

Lake	Type	TIHeLM	nEQR TIHeLM	*C*_max_ (m)	nEQR *C*_max_	HeLM	Ecological status
Kourna	GR-DNL	7.163	0.989	13.20	1.000	0.995	High
Feneos	GR-DNL	7.322	0.917	12.80	1.000	0.959	High
Megali Prespa	GR-DNL	7.483	0.847	7.00	0.681	0.764	Good
Trichonida	GR-DNL	7.694	0.740	10.00	0.773	0.757	Good
Amvrakia	GR-DNL	7.497	0.841	6.60	0.668	0.755	Good
Vegoritida	GR-DNL	7.579	0.807	7.67	0.701	0.754	Good
Volvi	GR-DNL	7.444	0.864	3.87	0.528	0.696	Good
Yliki	GR-DNL	7.439	0.866	3.80	0.519	0.693	Good
Paralimni	GR-SNL	7.141	1.000	6.80	1.000	1.000	High
Doirani	GR-SNL	7.783	0.687	4.67	0.848	0.768	Good
Mikri Prespa	GR-SNL	8.389	0.478	5.60	0.947	0.712	Good
Kastoria	GR-SNL	8.119	0.548	4.40	0.820	0.684	Good
Lysimachia	GR-SNL	7.958	0.593	3.40	0.576	0.585	Moderate
Ozeros	GR-SNL	8.788	0.379	3.10	0.524	0.452	Moderate
Zazari	GR-SNL	8.737	0.392	1.40	0.240	0.316	Poor
Pamvotida	GR-SNL	9.068	0.307	0.53	0.092	0.199	Bad

*GR-DNL* Greek deep natural lakes, *GR-SNL* Greek shallow natural lakes

### Evaluation of HeLM method’s performance

**Fig. 2:**
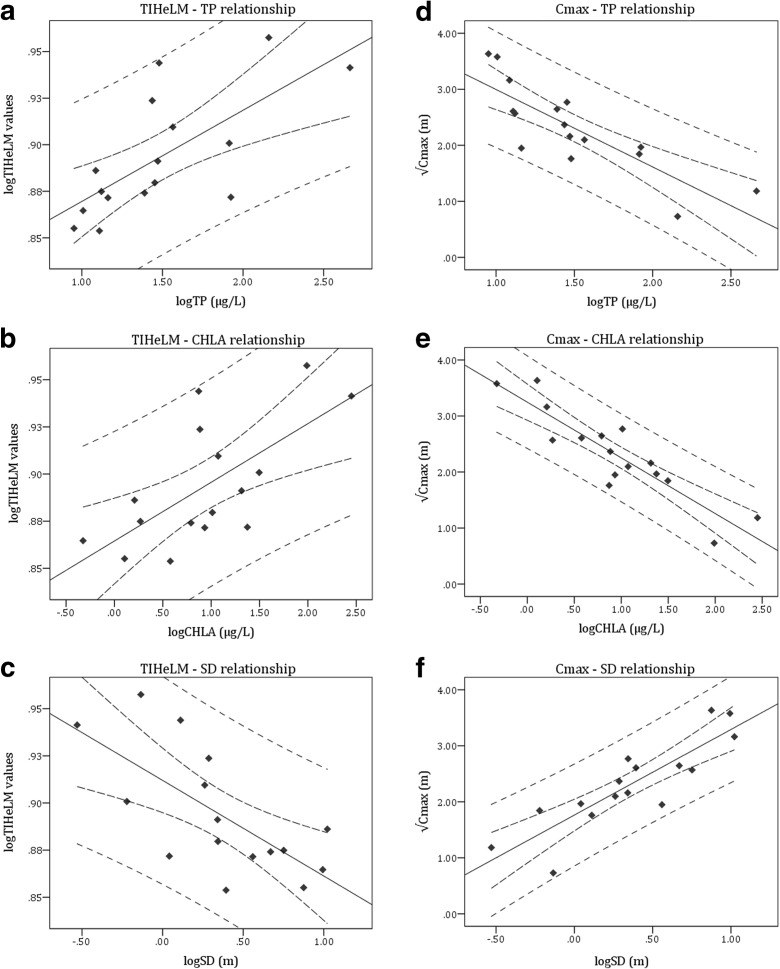
Linear relationships between the metrics TIHeLM (**a**–**c**) and *C*_max_ (**e**–**g**) and the pressure indicators total phosphorus (TP), chlorophyll *a* (CHLA), and Secchi depth (SD). Best linear fits, limits of 95% confidence, and prediction limits are shown. Best linear fits’ equations and coefficients can be seen at Table 6

**Fig. 3:**
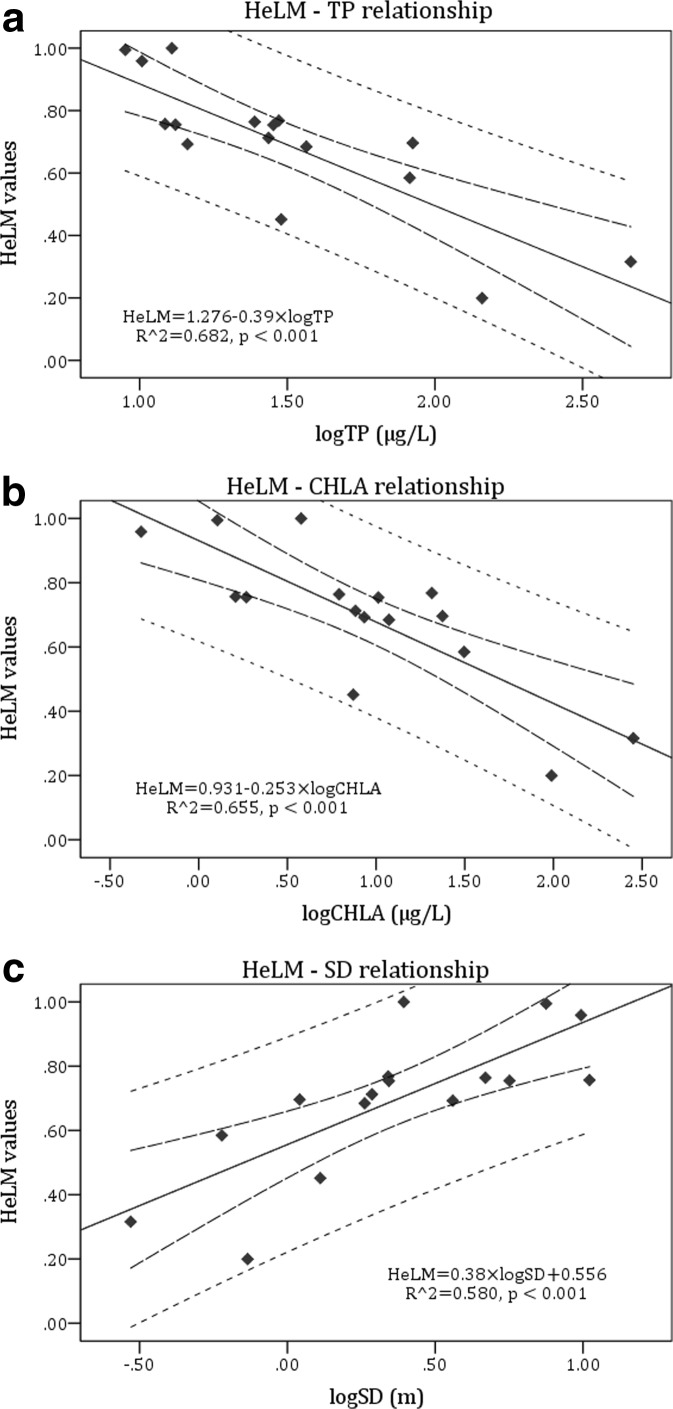
Linear relationships between final HeLM values and the pressure indicators total phosphorus (TP) (**a**), chlorophyll *a* (CHLA) (**b**), and Secchi depth (SD) (**c**). Best linear fits, limits of 95% confidence, and prediction limits are shown. Best linear fits’ equations and coefficients can be seen at Table 6

All linear regression models between the TIHeLM, *C*_max_, and HeLM values and the three pressure indicators were found significant at the 0.01 level, with coefficient of determination (*R*^2^) higher than 0.3 (the lower value was 0.454) and Pearson’s correlation coefficient (*R*) higher than 0.5 (the lower value was 0.674) (Table 6). Specifically, the TIHeLM metric showed a relatively high positive correlation with TP and CHLA, and an equal negative correlation with SD (Fig. 2 and Table 6). On the other hand, *C*_max_ metric showed a high negative correlation with TP and CHLA and an equal positive one with SD (Fig. 2 and Table 6). More importantly, final HeLM values showed a high negative correlation with TP and CHLA, and a high positive one with SD (Fig. 3 and Table 6). The multivariate regression analysis was also significant at the 0.01 level and with high coefficient of determination (*R*^2^) and Pearson’s correlation coefficient (*R*) (Table 6). Best linear fits, limits of 95% confidence, and prediction limits are shown in Figs. 2 and 3.

**Table 6:** Overview of the relationships between HeLM metrics (TIHeLM and *C*_max_) and HeLM final values and pressure indicator values (total phosphorus concentration, TP; chlorophyll *a* concentration, CHLA; and Secchi depth, SD), after linear regression and multivariate regression analysis

Relationship	*n*	*R* ^2^	*R*	*p*	Regression equation
TIHeLM-TP	16	0.494	0.703	0.002	log*TIHeLM* = 0.049 × log *TP* + 0.821
TIHeLM-CHLA	16	0.454	0.674	0.004	log*TIHeLM* = 0.031 × log *CHLA* + 0.865
TIHeLM-SD	16	0.475	− 0.689	0.003	log*TIHeLM* = 0.912 − 0.051 × log *SD*
*C*_max_-TP	16	0.686	− 0.828	< 0.001	$\sqrt{Cmax}=4.379-1.383\times \log TP\kern0.5em$
*C*_max_-CHLA	16	0.808	− 0.899	< 0.001	$\sqrt{Cmax}=3.247-0.994\times \log CHLA\kern0.5em$
*C*_max_-SD	16	0.751	0.867	< 0.001	$\sqrt{Cmax}=1.528\times \log SD+1.764\kern0.5em$
HeLM-TP	16	0.682	− 0.826	< 0.001	*HeLM* = 1.276 − 0.39 × log *TP*
HeLM-CHLA	16	0.655	− 0.809	< 0.001	*HeLM* = 0.931 − 0.253 × log *CHLA*
HeLM-SD	16	0.580	0.762	< 0.001	*HeLM* = 0.38 × log *SD* + 0.556
HeLM-TP, CHLA, and SD	16	0.694	− 0.833	0.002	*HeLM* = 1.256 − 0.29 × log *TP* − 0.112 × log *CHLA* − 0.068 × log *SD*

The coefficient of determination (*R*^2^), Pearson’s correlation coefficient (*R*), and the *p* value of significance are given for each regression

**Fig. 4:**
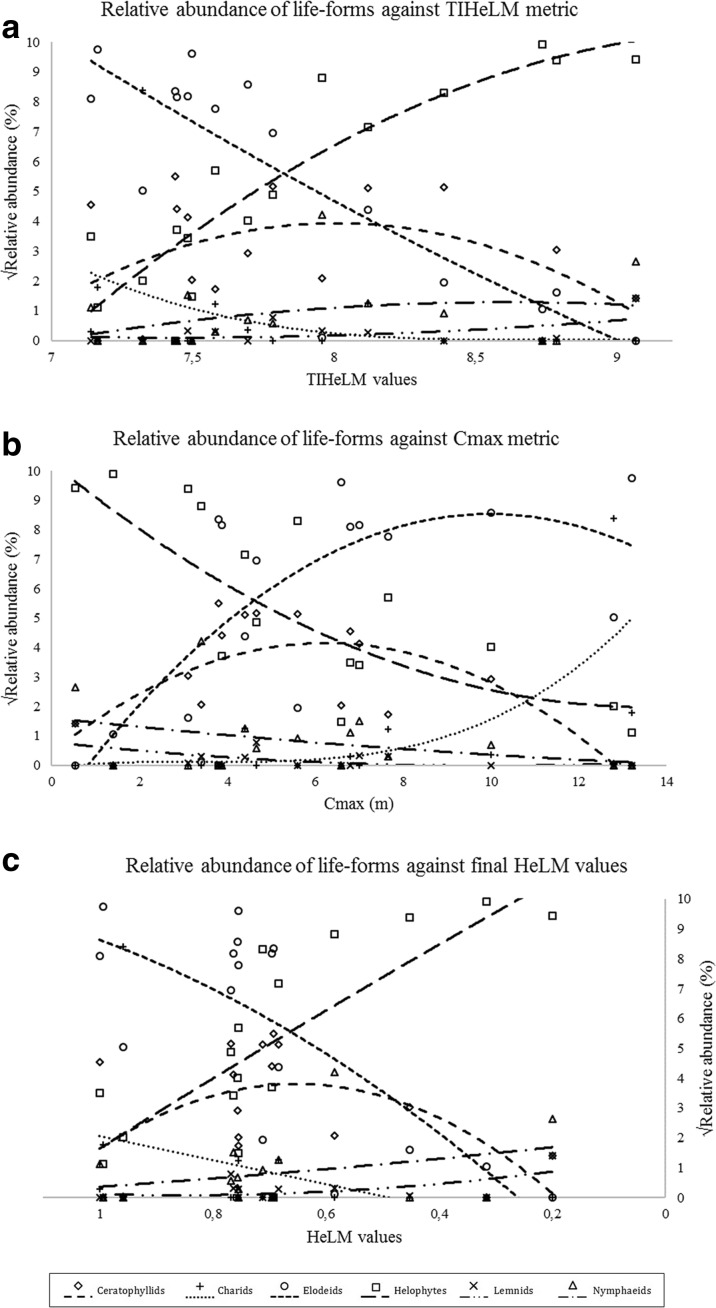
Scatterplots between TIHeLM (**a**), *C*_max_ (**b**), and final HeLM values (**c**) and the calculated relative abundance (square-root-transformed) of different life-forms of macrophytes in the studied lakes. The lines represent polynomial adjustments

Figure 4 represents the changes in relative abundance of different life-forms of macrophytes over the range of values of both taxonomic composition metric (TIHeLM) and abundance metric (*C*_max_), as well as the final HeLM assessment method values. In all three cases, a clear trend was observed of elodeid-dominated macrophytic communities at high ecological status lakes being replaced by helophyte-dominated macrophytic communities at lakes of lower ecological status classes. Charids were also quite commonly found in lakes at higher classes, and they were absent in lakes at lower classes. On the other hand, lemnids and nymphaeids showed a slight increasing trend towards lakes at lower classes of ecological status.

## Discussion

### Development of the HeLM assessment method

Our results show that HeLM assessment method is a fully WFD-compliant assessment method, able to classify the Greek lakes according to their ecological status. Currently, 17 MS have developed, intercalibrated, and harmonized their methods for assessing the ecological status of lakes based on aquatic macrophytes (European Commission 2013; Poikane et al. 2011). All these MS, without exception, belong to the Central and Northern European regions, whereas there is still not a fully harmonized method in Mediterranean region (Poikane et al. 2011, 2015). In these methods, MS follow three approaches with regard to the assessment of macrophyte taxonomic composition. The first is based on the relative abundance of sensitive and/or tolerant taxa [e.g., Dutch method (Coops et al. 2007), Norwegian method (Mjelde 2007), German method (Schaumburg et al. 2004)], the second on diversity indices [e.g., Polish method (Portielje et al. 2014), British method (Willby et al. 2009)], and the third approach on trophic scores of taxa [e.g., Swedish method (Ecke 2007), Austrian method (Pall and Moser 2009)]. In regard to abundance, maximum colonization depth (*C*_max_) is commonly used in the assessment methods of many other MS [e.g., Irish method (Free et al. 2006), Austrian method (Pall and Moser 2009), German method (Schaumburg et al. 2004), Danish method (Sondergaard et al. 2010)]. Based on the results of our tests that applied the rationale of Dudley et al. (2011) and Kolada et al. (2011, 2014), *C*_max_ and a trophic score index were chosen to become the constituent metrics of HeLM assessment method.

Another important issue we had to address during the development of a water ecological status assessment method was the establishment of reference conditions and ecological status class boundaries. In the HeLM method, potential reference sites were selected on the basis of pressure indicators and ecological criteria which present the potential anthropogenic effect in the catchment area of the lakes. These criteria were also recommended by REFCOND (European Commission 2003b) and were among the ones that are commonly used in other published assessment methods (Pahissa et al. 2015; Pall et al. 2016; Poikane et al. 2015; Portielje et al. 2014, etc.). Reference conditions for European lakes have been mostly developed on the basis of phytoplankton, with accepted lakes having annual TP concentrations up to 35 μg/L and annual CHLA concentrations up to 5 μg/L (Cardoso et al. 2007; Carvalho et al. 2008; McElarney and Rippey 2009; Toth et al. 2008). Thus, the thresholds set for reference criteria in HeLM method are consistent with those found in assessment methods of other MS (Hellsten et al. 2014; Portielje et al. 2014). Ecological status class boundaries were set following the recommendations of REFCOND (European Commission 2003b) and the work done in Mediterranean region (Pahissa et al. 2015). The resulted values are also comparable with the boundary values set in other MS (Hellsten et al. 2014; Portielje et al. 2014). However, for their final refinement, more data concerning changes in species compositions in Mediterranean lakes may be required.

### Application and evaluation of the HeLM method’s performance

TIHeLM and *C*_max_ independently do not always result in the same ecological status. There were cases that *C*_max_ classified the lake to a lower class than TIHeLM and vice versa. This can be attributed to the different time of response of the two metrics to changes in water quality. Taxonomic composition of macrophytes has been found to respond relatively slow to changes of trophic conditions, requiring some years to adapt to the new conditions within a lake (Jeppesen et al. 2005; Mehner et al. 2008; Melzer 1999; Robertson et al. 2000). On the other hand, *C*_max_ seems to adapt much faster to changes of trophic conditions (Asplund and Cook 1997; Mehner et al. 2008; Robertson et al. 2000; van den Berg et al. 1999). For this reason, it is important for an assessment method to cover different aspects of macrophytic vegetation, with the inclusion of short-time as well as long-time reacting components to eutrophication and re-oligotrophication processes (Pall and Moser 2009; Portielje et al. 2014; Schaumburg et al. 2004). Furthermore, calculation of the spatial variability of TIHeLM among transects within each lake can be a valuable tool for the identification of point sources of water pollution and the prevalence of different levels of ecological status in different sub-bodies of a lake (Brazner et al. 2007; Niemi et al. 2004). For example, in our data set, around half of Lysimachia lake showed a high level of degradation due to eutrophication and general degradation pressures, whereas the other half that receives water from the nearby lake Trichonida (Avramidis et al. 2013) did not.

The performance of the HeLM assessment method was tested by exploring the relationships of its constituent metrics with the most common eutrophication pressure indicators. The data set that was used for the above-mentioned test covered the full nutrient-pressure gradient that can be found in Greek natural lakes, so as to evaluate the reliability of the method to classify water bodies in all different classes of ecological status. The majority of the sites in the data set used were within the confidence limits (95%) of the assessment model, while all of them were within its prediction limits for all three pressure indicators. The final HeLM values were found to describe eutrophication pressures strongly and with high significance. For example, the coefficient of determination (*R*^2^) in the correlation with TP was found at *R*^2^ = 0.682. This value is among the highest values found in assessment systems of other MS (e.g., Finland TP, *R*^2^ = 0.29; Ireland TP, *R*^2^ = 0.56; Norway TP, *R*^2^ = 0.65; United Kingdom TP, *R*^2^ = 0.48; Germany TP, *R*^2^ = 0.50; France TP, *R*^2^ = 0.33; Austria TP, *R*^2^ = 0.27) (Hellsten et al. 2014; Pall et al. 2014; Portielje et al. 2014). Furthermore, all values of determination coefficient (*R*^2^) and Pearson’s correlation coefficient (*R*), for all correlations between HeLM’s metrics and final values and pressure indicator values, over-exceeded the thresholds of *R*^2^ > 0.30 and *R* > 0.55 proposed for well-performing metrics by Kolada et al. (2011). All the above are indicative of the effective performance of the HeLM assessment method in addressing eutrophication and general degradation pressures in Greek natural lakes.

Individual metric and final HeLM values were also found to discriminate quite well the lakes where elodeids and charids are dominating the macrophytic vegetation from the lakes where only helophytes, lemnids, and nymphaeids are present and thus the shift from a submerged macrophytic vegetation to an emergent one (Kolada 2014). This shift has been found to be strongly connected with a change from a transparent state of water to a turbid one, thus representing a water deterioration gradient (Hilt et al. 2010; Klosowskii et al. 2006; Kohler et al. 2010; Kolada 2014; Spoljar et al. 2017). Specifically, a great number of charophytes and elodeids are considered as indicative of high and good ecological status sites (Hellsten et al. 2014; Pall et al. 2014; Portielje et al. 2014), while for less than good status communities, the above-mentioned taxa are being replaced by more tolerant taxa, such as nymphaeids, lemnids, and helophytes (Kolada 2014; Pall et al. 2014; Penning et al. 2008a, b; Portielje et al. 2014; Toth et al. 2008).

### Limitations and future needs

The taxonomic composition metric TIHeLM used in the HeLM assessment method is a trophic score metric, calculated on the basis of species trophic scores (LTR). Since there is neither a current trophic scoring system for macrophytes in Greek lakes nor the amount of data necessary for the development of one, the calculation of the TIHeLM metric uses the scoring system resulted from the European intercalibration exercise (Hellsten et al. 2014; Kolada et al. 2011, 2014). Furthermore, new LTR values have been calculated by applying a regression (Kolada et al. 2011) for about half of the taxa recorded in our data set, as no LTR values existed for them. The continuous operation of Monitoring Networks by Mediterranean MS will provide more data considering TP optima for macrophytes in the Mediterranean region in the future; thus, the LTR values of taxa may be revised to represent more accurately their response to the trophic status of lakes.

An additional issue to be treated is that the development of the HeLM assessment method was based on a data set representing 16 lakes and a 3-year sampling period. Future sampling campaigns in the context of the monitoring network will provide additional data. As more data become available, the method needs to be checked and revised accordingly in subjects such as analysis of the uncertainty, temporal stability of metric values within reference sites, analysis of importance of the inclusion or not of specific groups of taxa in the metrics calculation, and readjustment of boundary locations according to changes in species composition.

Finally, an important issue for the future is the harmonization of the methods used in the Mediterranean region. Following the development efforts for the French macrophyte assessment method for lakes, IBML (Bertrin et al. 2016), Italian macrophyte method VL-MMI (Azella 2016), and the Spanish assessment method (CEDEX 2010a, b), all Mediterranean MS, should focus on the intercalibration of their adopted methods, in order to ensure that good ecological status represents the same level of ecological quality for all lakes in the Mediterranean region, consistent with WFD normative definitions (Poikane et al. 2011; Toth et al. 2008).

## Conclusions

The WFD-compliant HeLM assessment method for macrophytes in Mediterranean lakes in Greece was developed on the basis of two metrics concerning the taxonomic composition and abundance of aquatic macrophytes. According to the so far collected data from the Greek National Water Monitoring Network, the developed method depicts in a satisfactory manner the response of aquatic macrophytes to eutrophication and general degradation pressures. It is also able to discriminate lakes with macrophytic vegetation dominated by different life-forms. In the light of new data and under the requirements among other MS in the Mediterranean region, future additions and improvements in this assessment method may be required.

## Electronic supplementary material

ESM 1 (PDF 457 kb)
