# Supplementary material for: HeLM: a macrophyte-based method for monitoring and assessment of Greek lakes
Source: Environ Monit Assess. 2018 May 5;190(6):326. doi: 10.1007/s10661-018-6708-1 (PMC5937868; doi:10.1007/s10661-018-6708-1)

Electronic Supplementary Material

HeLM: A macrophyte-based method for monitoring and assessment of Greek Mediterranean lakes

Dimitrios Zervas [a,b,*], Vasiliki Tsiaoussi [a], Ioannis Tsiripidis [b]

[a] The Goulandris Natural History Museum - Greek Biotope / Wetland Centre, 14th km Thessaloniki-Mihaniona, P.O. Box 60394, Thermi 57001, Thessaloniki, Greece, e-mail: dzervas@ekby.gr

[b] Department of Botany, School of Biology, Aristotle University of Thessaloniki, GR-54124, Greece

Supplement 1. References used for the taxonomic identification of aquatic macrophyte taxa found in the 16 Greek lakes

**Category of organisms**	**Reference**
Angiosperms and pteridophytes	Ascherson, P., Graebner, P., 1959. Potamogetonaceae. Das Pflanzenreich, Regni vegetabilis conspectus, H. 81. Verlag von H.R. Engelmann (J. Cramer Weinheim Bergstrasse).
	Casper, S.J., Krausch, H.D., 1980, 1981. Pteridophyta und Anthophyta 1-2. Subwasserflora von Mitteleuropa, Bd 23-24. Gustav Fischer Verlag, Stuttgart, New York.
	Cook, C.D.K., 1990. Aquatic Plant Book. SPB Academic Publishing. The Hague, The Netherlands.
	Correll, D.S., Correll, H.B., 1972. Aquatic and wetland plants of Southwestern United States. Environmental Protection Agency, Washington.
	Crow, G.E., Hellquist, C.B., 2000. Aquatic and wetland plants of Northeastern North America. The University of Wisconsin Press, Wisconsin.
	Fasset, N.C., 1957. A manual of aquatic plants. 2nd ed. The University of Wisconsin Press, Wisconsin.
	Strid, A., Tan, K., 1997. Flora Hellenica vol. I. Koeltz Scientific Books edn. Königstein.
	Strid, A., Tan, K., 2002. Flora Hellenica vol. II. Ruggell.
	Tutin, T.G., Burges, N.A., Charter, A.O., Edmondson, J.R., Heywood, V.H., Moore, D.M., Valentine, D.H., Walters, S.M., Webb, D.A.E., 1993. Flora Europaea I. 2nd edn. Cambridge University Press, Cambridge, U.K.
	Tutin, T.G., Heywood, V.H., Burges, N.A., Moore, D.M., Valentine, D.H., Walters, S.M., Webb, D.A.E., 1968, 1972, 1976, 1980. Flora Europaea II-IV. Cambridge University Press, Cambridge, U.K.
Bryophytes	Pedrotti, C.C., 2001, 2006. Flora dei Muschi d' Italia. Parte I-II. Antonio Delfino Editore, Libro Universitario.
	Schumacker, R., Vana, J., 2005. Identification keys to the Liverworts and Hornworts of Europe and Macaronesia. 2nd ed. Sorus, Poznan.
Charophytes	Krause, W., 1997. Charales (Charophyceae). Subwasserflora von Mitteleuropa, Bd 18. Spektrum Akademischer Verlag, Heidelberg.
	Wood, R.D., Imahori, K., 1964. Iconograph of the Characeae. Verlag von J. Cramer, Weinheim.
	Wood, R.D., Imahori, K., 1965. Monograph of the Characeae. Verlag von J. Cramer, Weinheim.

Supplement 2. Scatter-plot between total phosphorus values (log-transformed) and the relative abundance (square-root transformed) of different biotic forms of aquatic macrophytes in Greek lakes (pressure-response curves). The lines represent polynomial adjustments. Square area represents sites that belong to the 20-50μg/L TP-group, where the transition point from submerged-dominated (elodeids and charids) macrophytic communities to helophyte-dominated macrophytic communities occurs

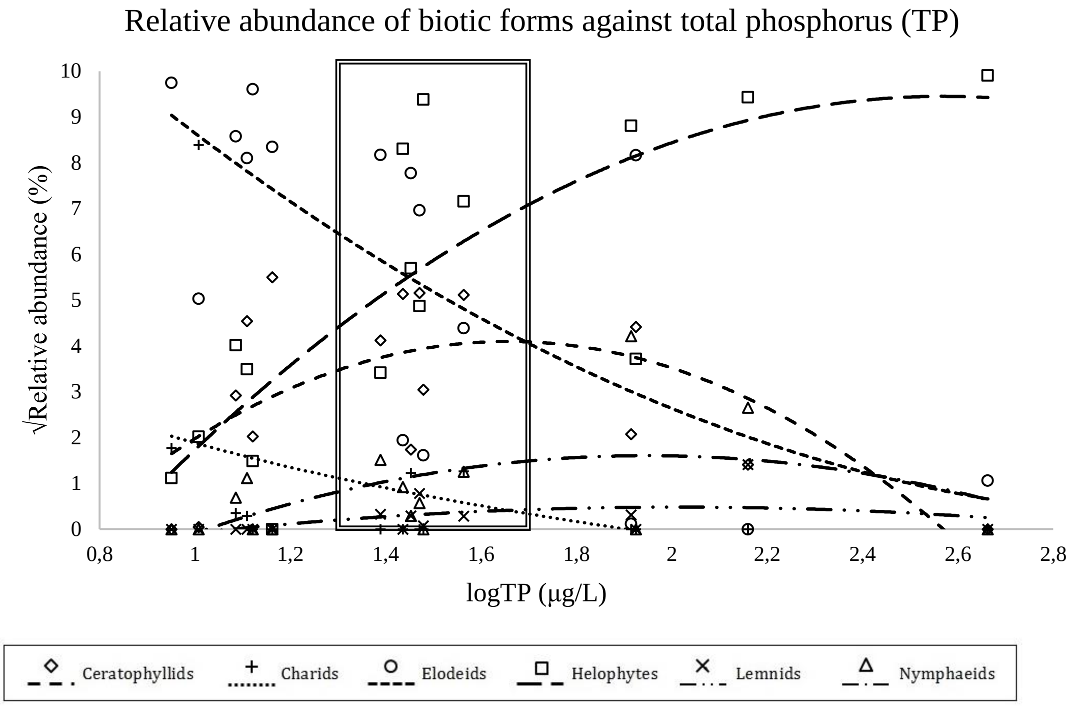

Supplement 3. Paired metric analysis of TIHeLM and Cmax metric values in Greek lakes, using regression lines against total phosphorus values (log-transformed). Square area represents sites that belong to the 20-50μg/L TP-group, that are found at the space before metrics cross-over point which is associated with moderate ecological status sites

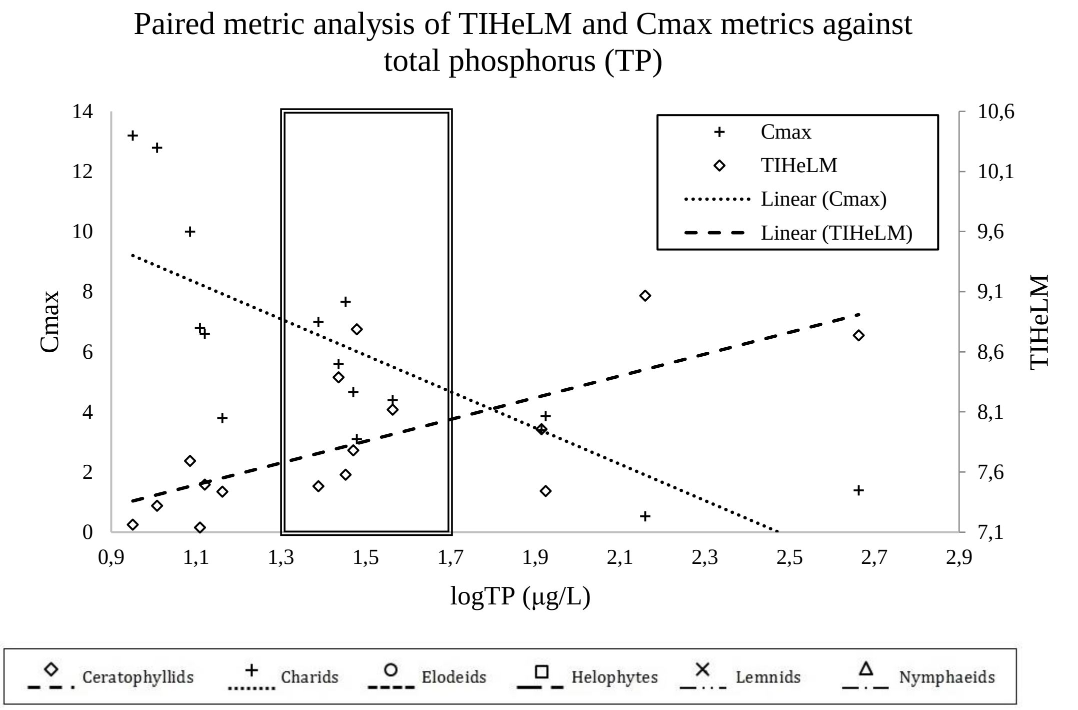

Supplement 4. Aquatic macrophyte taxa found in the 16 Greek lakes, along with their Lake Trophic Rank values (LTRs) as elaborated by Kolada et al. (2011, 2014). LTR values derived from the regression between all taxa LTR values and Ellenberg indicator values for nutrients are marked with asterisks

**Taxon Name**	**LTR**	**Taxon Name**	**LTR**
Agrostis stolonifera	*6.35	*Najas minor*	*4.96
Alisma gramineum	*4.96	*Nitella gracilis*	4.17
Alisma lanceolatum	*6.35	*Nitella hyalina*	3.81
Alisma plantago-aquatica	*10.54	*Nitella syncarpa*	3.81
Arundo donax	*7.75	*Nitellopsis obtusa*	6.13
Azolla filiculoides	*10.54	*Nuphar lutea*	7.05
Berula erecta	*7.75	*Nymphaea alba*	6.02
Bolboschoenus maritimus	9.14	*Nymphoides peltata*	7.76
Butomus umbellatus	8.73	*Paspalum dilatatum*	*9.14
Carex sp.	*4.96	*Paspalum distichum*	*9.14
Ceratophyllum demersum	7.82	*Persicaria amphibia*	8.07
Ceratophyllum submersum	7.85	*Phalaroides arundinacea*	*9.14
Chara aspera	4.70	*Phyla nodiflora*	*6.35
Chara corfuensis	6.03	*Phragmites australis*	*9.14
Chara globularis	6.80	*Potamogeton berchtoldii*	5.73
Chara hispida	4.48	*Potamogeton compressus*	5.43
Chara tomentosa	5.27	*Potamogeton crispus*	8.02
Chara vulgaris	6.53	*Potamogeton gramineus*	3.17
Elatine triandra	5.21	*Potamogeton lucens*	6.01
Eleocharis mitracarpa	*4.96	*Potamogeton nodosus*	*6.35
Eleocharis palustris	*4.96	*Potamogeton perfoliatus*	4.95
Eleocharis parvula	*6.35	*Potamogeton pusilus*	9.10
Elodea canadensis	7.42	*Potamogeton trichoides*	7.19
Epilobium lanceolatum	*3.56	*Ranunculus rionii*	3.81
Epilobium parviflorum	*7.75	*Ranunculus trichophyllus*	3.81
Filamentous macroalgae	8.78	*Rorippa amphibia*	*10.54
Hydrocharis morsus-ranae	7.09	*Rumex palustris*	*10.54
Iris pseudacorus	*9.14	*Salvinia natans*	*9.14
Juncus articulatus	*2.16	*Samolus valerandi*	*6.35
Juncus inflexus	*4.96	*Schoenoplectus lacustris*	*7.75
Juncus subnodulosus	*3.56	*Schoenoplectus litoralis*	*7.75
Juncus tenuis	*6.35	*Scirpoides holoschoenus*	*10.54
Lemna gibba	9.63	*Sparganium angustifolium*	2.69
Lemna minor	8.82	*Sparganium erectum*	*9.14
Ludwigia peploides	*4.96	*Sparganium neglectum*	*7.75
Lycopus europaeus	*9.14	*Spirodela polyrhiza*	9.57
Lysimachia vulgaris	*6.35	*Stuckenia pectinata*	8.64
Lythrum salicaria	*6.35	*Trapa natans*	*10.54
Mentha aquatica	*6.35	*Trichophorum cespitosum*	*0.77
Mentha pulegium	*3.56	*Typha angustifolia*	*9.14
Fontinalis antipyretica	5.48	*Typha domingensis*	*10.54
Myriophyllum spicatum	7.30	*Typha latifolia*	*10.54
Myriophyllum verticillatum	5.74	*Utricularia vulgaris*	3.86
Najas gracillima	*10.54	*Vallisneria spiralis*	*7.75
Najas graminea	*6.35	*Vitex agnus-castus*	*3.56
Najas marina	6.78	*Zannichellia pedunculata*	9.53

Supplement 5. Box and whiskers plots of mean annual values of total phosphorus (TP), mean summer values of chlorophyll-a (CHLA), mean summer values of Secchi depth (SD), artificial land use (ALU), intensive agriculture (IA), natural and semi-natural land use cover (NASN) and population density (PD) in reference and non-reference lakes of the two national types (GR-DNL and GR-SNL). Boxes represent 25th-75th percentiles, horizontal lines within are medians and range bars are minimum-maximum values

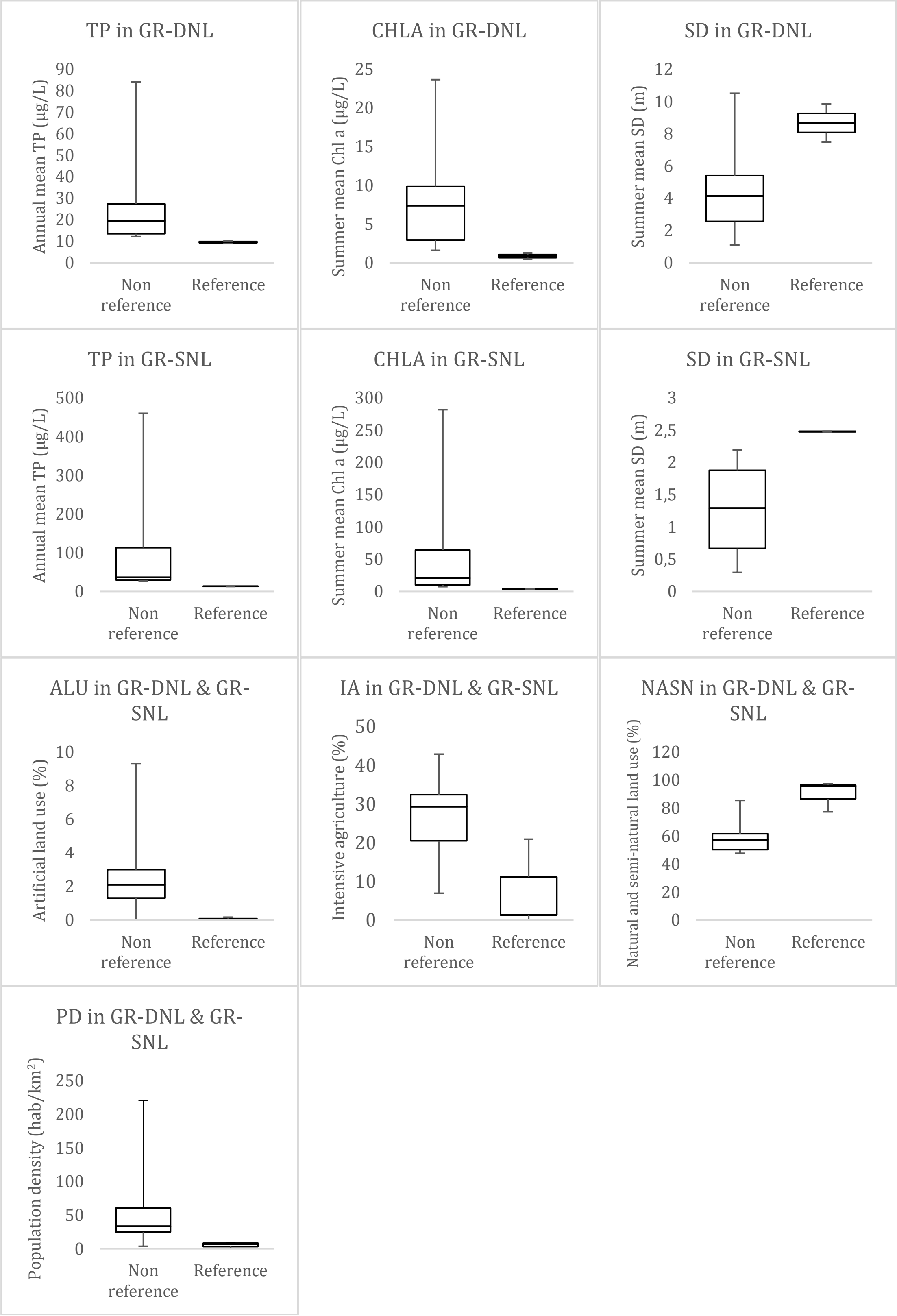

Supplement 6. TIHeLM metric transect values variation within each lake (see Table 1 for lake abbreviations and number of transects). Boxes represent 25th-75th percentiles, horizontal lines within are medians, range bars indicate minimum and maximum values (95% distribution), open circles represent outliers and asterisks extreme outliers

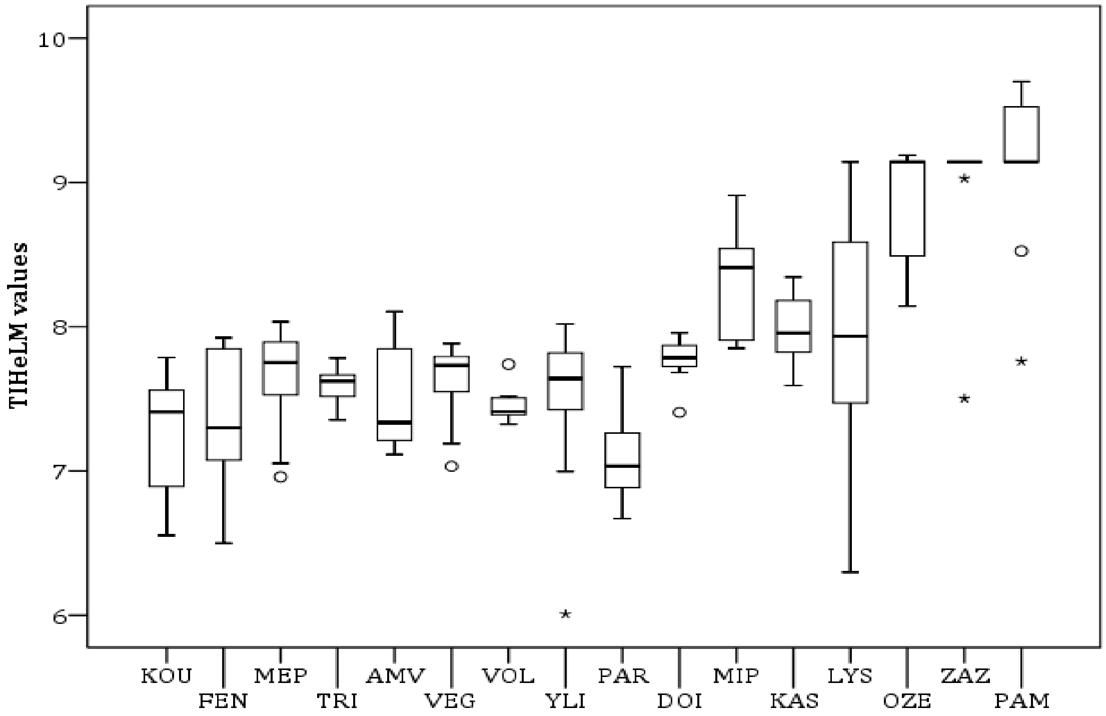

Supplement: Supplementary file 1 — (PDF 457 kb) [file 10661_2018_6708_MOESM1_ESM.pdf]
